# Detection of DNA from *Leishmania (Viannia)*: Accuracy of Polymerase Chain Reaction for the Diagnosis of Cutaneous Leishmaniasis

**DOI:** 10.1371/journal.pone.0062473

**Published:** 2013-07-05

**Authors:** Herintha Coeto Neitzke-Abreu, Mateus Sabaini Venazzi, Marcos Vinicius Zandonadi Bernal, Kárin Rosi Reinhold-Castro, Fernanda Vagetti, Camila Alves Mota, Naielly Rodrigues Silva, Sandra Mara Alessi Aristides, Thaís Gomes Verzignassi Silveira, Maria Valdrinez Campana Lonardoni

**Affiliations:** 1 Health Sciences Postgraduate Program (PCS), Universidade Estadual de Maringá, Maringá, State of Paraná, Brazil; 2 Department of Clinical Analysis and Biomedicine (DAB), Universidade Estadual de Maringá, Maringá, State of Paraná, Brazil; Technion-Israel Institute of Technology Haifa 32000 Israel., Israel

## Abstract

Cutaneous leishmaniasis (CL) can occur in skin and mucosa, causing disfiguring lesions. The laboratory diagnosis of CL involves immunological methods and optical detection of the parasite, al of which have limitations. There is a need for more effective diagnostic methods for CL which wil allow treatment to be initiated more promptly in order to help prevent the development of severe forms of mucosal disease, and to estimate the prognosis of the infection. The polymerase chain reaction (PCR) has been widely used to diagnose CL, because of its higher sensitivity. This study estimated the accuracy and compared PCRs of samples from lesion scarification (PCR-L) and blood sample-enriched leukocytes (PCR-B) with three conventional diagnostic techniques: parasite direct search (DS), Montenegro skin test (MST), and indirect immunofluorescence reaction (IIF). The study included 276 patients under suspicion of CL. We conducted a cross-sectional study, in which patients were selected by convenience sampling. We used MP3H/MP1L primers to generate a *Leishmania (Viannia)* (minicircle kDNA) fragment of 70-bp. Of 106 patients with CL, 83.87%, 51.67%, 64.52%, 85.71%, or 96.10% tested positive by PCR-L, PCR-B, DS, IIF, or MST, respectively. Five patients tested positive only by PCR-L, and two other patients only by PCR-B. PCR-L is indicated for use in patients with chronic lesions or *Leishmania* reinfection, which may progress to mucosal lesion. PCR-B is indicated for use in patients with negative results in conventional tests or for patients with no apparent lesion. PCR is not only useful in diagnosing CL but also helps to identify the infecting species.

## Introduction

Leishmaniases are protozoonoses that are found in 88 countries, with approximately 14 million infected people [1]. Some forms of leishmaniasis may cause destructive and disabling injuries, which can lead to death [2].

Cutaneous leishmaniasis (CL) is recognized by the cutaneous form. In Brazil, CL cases are mainly due to *Leishmania (Viannia) braziliensis*, which causes lesions that if left untreated might result in the mucosal form, which is characterized by disfiguring lesions [2] that can destroy cartilage [3]. Accurate and early diagnosis is important in cases of CL caused by *L. (V.) braziliensis*, and involves laboratory tests to detect the parasite (direct search, culture, animal inoculation, and histopathology) and immunodiagnostic techniques (detection of cellular immune response, antibody, and immune-complexes antigen) [4].

The standard diagnosis of CL is accomplished by demonstrating the presence of the parasite [2], although the methods that are currently used have limitations [5]. Histopathology, an invasive technique, and direct parasite search (DS) have low positivity; they depend on the number of parasites in a sample, and have limited application in patients with old lesions or patients who show no lesions [2].

Methods involving isolation of the parasite in culture and inoculation in animals can be used; however, their performance depends on the species of *Leishmania,* and the culture media may become heavily contaminated with bacteria [6]. The use of experimental animals is complicated by the long period of time required for the lesion to evolve [2] and the ethical aspects involved. Therefore, culture and animal inoculation are not practical for routine laboratory diagnosis.

Serological tests for antibodies are often used to diagnose CL. Although they are easy to perform, serological methods show higher rates of negativity in patients with only one lesion or with lesions less than six months old [2].

The Montenegro skin test (MST) has been reported to show high rates of positivity [7], [8], [9]. However, in HIV-positive patients, MST shows a false negative result due to the lack of a cellular immune response against parasite antigens [2]. Furthermore, MST is an invasive technique, may give a positive result in latent infections, and does not distinguish a former from a current infection [8], [10].

The presence of different *Leishmania* species with similar clinical characteristics in endemic areas requires the development of more accurate and sensitive laboratory methodologies to identify these species, in order to assess the CL prognosis and allow the selection of an appropriate therapy [11], [12]. Species identification also contributes to better understanding of CL epidemiology [13]. The Polymerase Chain Reaction (PCR), because of its high sensitivity, has been widely used to detect and identify *Leishmania* species in different clinical samples [8], [9], [14], [15], [16], [17], [18].

Although this disease has been extensively studied, some aspects remain unknown and still under discussion. It is necessary to improve the diagnosis of CL by identifying the species involved, in order to provide more rapid, safer, and more appropriate treatment, as well as to determine the prognosis of the infection. The aim of this study was to estimate the accuracy of PCR in biological samples obtained by scarification of lesions and from peripheral-blood leukocytes, comparing this with other conventional techniques used to diagnose CL, because, although DS is the reference technique for CL diagnosis, its diagnostic value remains uncertain.

## Methods

### Ethical Statement

The present study received approval from the Permanent Committee for Ethics in Research involving Humans (Process No. 533/2009) of the Universidade Estadual de Maringá. All participants were informed about the importance and objectives of the study, and were assured of both anonymity and confidentiality. We obtained written informed consent from patients who agreed to participate, or from the parents or guardians of patients who were minors. All procedures involving humans were conducted according to protocols approved by the National Health Council of the Brazilian Ministry of Health (Resolution No. 196/1996).

### Patient Selection

**Figure 1:**
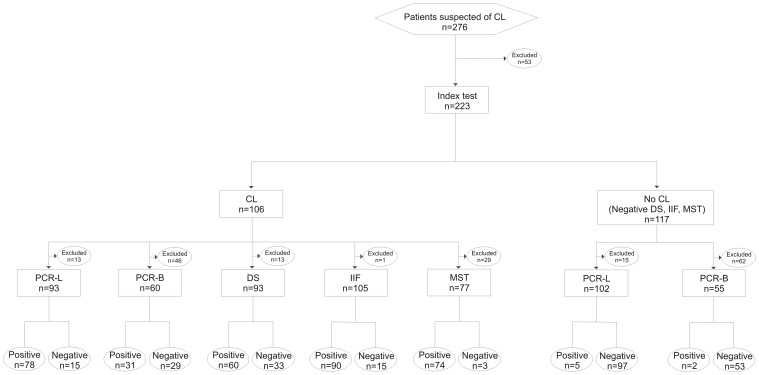
Flow diagram of patients to estimate the accuracy of PCR in the diagnosis of CL.

The study included 276 patients under suspicion of CL, who were referred by the 15th Regional Health Unit of Paraná (Brazil) to the Laboratory for Teaching and Research in Clinical Analysis of the Universidade Estadual de Maringá (UEM), which is the reference laboratory of the Brazilian Ministry of Health for diagnosis of CL, during the period from June 2010 through November 2011. The study used a cross-sectional desing, in which patients were selected by convenience sampling. We excluded 53 patients for whom at least one of the PCR analyses (lesion and/or blood) had not been performed (Figure 1). For all patients at least one conventional test and one of the molecular tests were performed. The patients completed a questionnaire that included clinically and epidemiologically relevant information. After the laboratory tests were performed, the patients returned to their referring physician for treatment.

### Conventional Laboratory Diagnosis

The methods were performed by a trained and qualified professional.

#### Direct parasite search (DS)

Material from the edge of the lesion was obtained by scarification after asepsis, with a non-metallic DNA spatula (previously treated with 1.5% sodium hypochlorite solution for 15 min and flame-sterilized). For detection of amastigotes of *Leishmania* sp., the glass slides containing the samples were fixed with methanol, stained with Giemsa, and analyzed by optical microscopy [8].

#### Indirect Immunofluorescence (IIF)

Venous blood was used for detection of IgG anti-*Leishmania*. The antigens used were promastigotes of *L. (V.) braziliensis* (MHOM/BR/1987/M11272), and conjugated anti-human immunoglobulin G - fluorescein (Biolab-Mérieux, Brazil). Titers ≥40 were considered positive [19].

#### Montenegro skin test (MST)

0.1 mL of antigen was injected intradermally in the arm of each patient. The site was evaluated after 48 h, and the presence of a papule ≥5 mm in diameter was considered a positive result [3], [8]. All samples were collected prior to administration of the MST.

### Molecular Diagnostics

#### Obtaining lesion DNA

Material from the edge of the lesion obtained by scarification was placed in two tubes free of RNAses and DNAses, containing 50 µL of STE buffer (10 mM TRIS, 1 mM EDTA, 0.1 M NaCl, pH 8.0). Samples were incubated at 95°C for 30 min in a PC Thermocycler (Biometra, Germany), centrifuged at 13,000 *g* for 1 min, and the supernatant was stored at −18°C until PCR (PCR-L) [8].

#### DNA extraction from blood

3 mL of venous blood was used to obtain leukocytes: blood was added to 1 mL of 10% EDTA and 6% Dextran (T500) [20]. The supernatant was removed after 1 h, divided into two tubes, and centrifuged at 200 *g* for 10 min. The pellet was washed with 0.15 M NaCl, centrifuged at 200 *g* for 10 min, resuspended with 125 µL of 0.15 M NaCl and 125 µL of ACD (25 mM citric acid, 50 mM sodium citrate, 81 mM D-glucose) and stored at −18°C for DNA extraction and PCR (PCR-B). Leukocyte-enriched samples were washed with PBS (saline solution buffered with 10 mM sodium phosphate, 0.15 M NaCl, pH 7.2) and centrifuged at 3,500 *g* for 15 min. DNA was extracted by the guanidine-phenol method [9] and resuspended in 50 µL of TE buffer (10 mM TRIS, 1 mM EDTA, pH 8.0). A positive control (blood from individuals without CL plus 10^4^ *L. (V.) braziliensis* promastigotes) and a negative control (blood from individuals without CL) were included.

#### PCR for amplification of *Leishmania* DNA

The primers MP3H (5'-GAA TTC GGT TGT CGG ATG C-3') and MP1L (5'-ACA TAC GCC TCC CTC TGC TG-3') [21] were used to amplify a 70-bp fragment from the kDNA minicircles of subgenus *Leishmania (Viannia)*. The reaction mixture (final volume 25 µL) was composed of 1 µM of each primer (Invitrogen Life Technologies, São Paulo, Brazil), 1.5 mM MgCl_2_, 1× enzyme buffer, 0.2 mM dNTP (Invitrogen, Carlsbad, CA, USA), 1 U Taq DNA polymerase (Invitrogen, Carlsbad, CA, USA), and 5 µL of DNA obtained from a lesion or 2 µL of DNA extracted from blood. DNA amplification was carried out in a PC Thermocycler (Biometra, Germany) at 95°C for 5 min, followed by 35 cycles: 1.5 min at 95°C, 1.5 min at 55°C, and 2 min at 72°C; finally for 10 min at 72°C. The product was kept at 4°C until analysis. Ten microliters of amplified products was submitted to electrophoresis in 3% agarose gel (Invitrogen, Paisley, Scotland, UK), stained with 0,1 µg/mL ethidium bromide, at 10–15 V/cm. A positive control [1 pg of *L. (V.) braziliensis* DNA] and a negative control (water) were added. The presence of bands was observed in a transilluminator (Macro Vue™ UV-20, Hoefer).

#### Internal amplification control

The samples (blood and lesion) with negative PCR results for *Leishmania* were submitted to PCR for the presence of inhibitors. Specific primers for the human β-globin were used, which amplify a fragment of 268-bp (GH20∶5'-GAA GAG CCA AGG ACA GGT AC-3', and PC04∶5'-CAA CTT CAT CCA CGT TCA CC-3') [22]. The analytical sensitivity was assessed using a sample of DNA from the lesion, measured by a Qubit™ Fluorometer Kit (Invitrogen, USA), and serially diluted (500 pg, 50 pg, 5 pg, 500 fg, 50 fg, 5 fg). The reaction mixture (final volume 25 µL) was composed of 1 mM of each primer (Invitrogen Life Technologies, São Paulo, Brazil), 3 mM MgCl_2_, 1× enzyme buffer, 0.2 mM dNTP (Invitrogen, Carlsbad, CA, USA), 1 U of Taq DNA polymerase (Invitrogen Life Technologies, São Paulo, Brazil) and 2 µL of DNA. The PCR was carried out in a PC Thermocycler (Biometra, Germany) by 40 cycles: 1 min at 95°C, 1 min at 55°C, and 2 min at 72°C; finally for 10 min at 72°C. The product was kept at 4°C until analysis. Ten microliters of amplified products was submitted to electrophoresis in 2% agarose gel (Invitrogen, Paisley, Scotland, UK), stained with ethidium bromide. The bands were observed in a transilluminator (Macro Vue™ UV-20, Hoefer).

#### Statistical analysis

The PCR-L and PCR-B results were compared with conventional methods using McNemar's test with the program Statistica 7.1, considering p≤0.05 to be significant. The proportions were analyzed using a Mid-p exact test OpenEpi version 2.3, with a confidence interval of 95% (95% CI). Sensitivity (S), specificity (Sp), positive predictive value (PPV), and negative predictive value (NPV) were determined for the DS test.

## Results

We studied 223 patients who were under suspicion of CL and residents of endemic areas; 70.40% were men and 29.60% were women. The majority (82.51%) were more than 30 years of age, and lived in urban areas (83.52%); however, the infections occurred mainly in patients from rural areas (62.42%).

In the 223 patients suspected of CL, 91.48% had skin lesions, in most cases a single lesion (65.97%). The evolution of lesions over time ranged from 1 week to 10 years, and the majority (54.08%) up to three months. Mucosal changes occurred in 8.52% of patients, and their evolution over time ranged from 2 months to 20 years, the majority within 2 years. All 223 patients were tested by PCR methods (lesion and/or blood), but not all patients were given one of the three conventional tests.

PCR-L was positive in 42.56% (83/195) and PCR-B 28.70% (33/115). DS was positive in 30.46% (60/197), IIF in 40.91% (90/220), and MST in 44.31% (74/167). The IIF resulted in titers ≤320 and MST papule diameter ≤30 mm. PCR-L showed higher positivity than DS (p = 0.0001), IIF (p = 0.0203), and showed no significant difference compared to MST (p = 0.1208). PCR-B detected fewer positive samples than DS (p = 0.0003), IIF (p = 0.0058), and MST (p = 0.0158).

**Figure 2:**
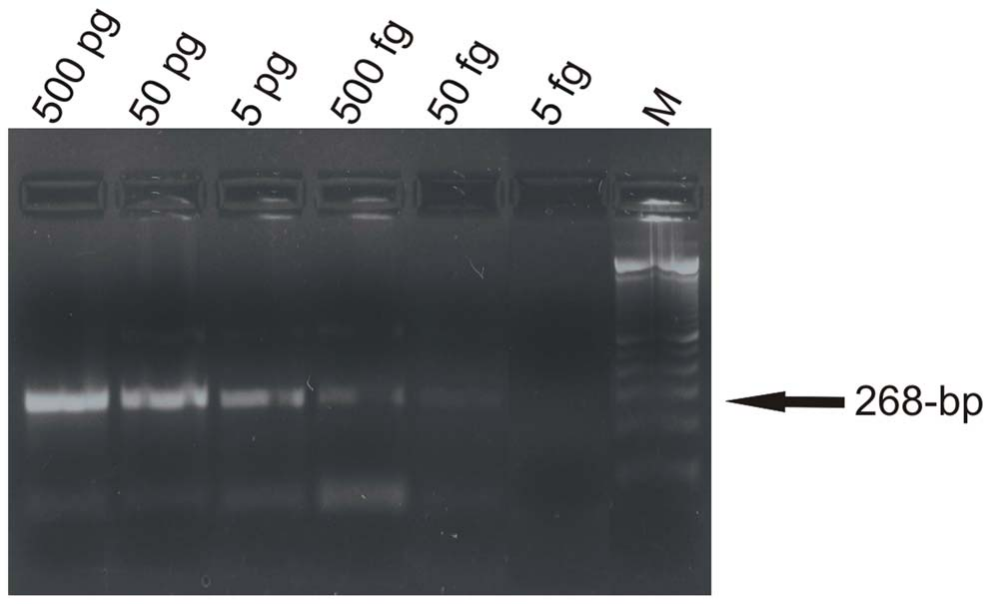
PCR analytical sensitivity, showing 268-bp fragment of human β-globin gene region. M, 100-bp molecular marker (Invitrogen Life Technologies, São Paulo, Brazil).

PCR carried out with GH20 and PC04 primers showed a detection limit of 50 fg DNA. All negative PCR samples for *Leishmania* were positive in the PCR for internal amplification control, excluding PCR inhibitors in these samples (Figure 2).

In the 223 patients, 106 (47.53%) were diagnosed positive for CL. Those individuals with at least one conventional positive test (DS, IIF, MST) were considered to be infected with *Leishmania*. In these patients, PCR-L was positive in 83.87% (78/93) and PCR-B in 51.67% (31/60); whereas DS was positive in 64.52% (60/93), IIF in 85.71% (90/105), and MST in 96.10% (74/77). PCR-L showed a higher positivity rate than DS (p = 0.0000), and lower than IIF (p = 0.0000) and MST (p = 0.0000). PCR-B showed no significant difference from DS (p = 0.2300), and detected fewer positive samples than IIF (p = 0.0000) and MST (p = 0.0002).

PCR-L was positive in 100% (95% CI; 95.05–100.00) of patients with a positive DS, in 54.55% (95% CI; 37.53–70.79) with a negative DS, and in 69.23% (95% CI; 41.30–89.37) with a negative IIF. PCR-B was positive in 65.63% (95% CI; 48.12–80.42) of patients with a positive DS, in 13.33% (95% CI; 2.30–37.52) with a negative DS, and in 60.00% (95% CI; 18.24–92.65) with a negative IIF (Table 1).

**Table 1:** PCR-L and PCR-B results for 106 patients with CL, according to conventional test results.

Indexes		PCR-L		PCR-B	
		Pos	Neg	Pos	Neg
**DS**	**Pos (n = 60)**	59/59	0/59	21/32	11/32
		100.00; 95.05–100.00	0.00; 0.00–4.95	65.63; 48.12–80.42	34.38; 19.58–51.88
	**Neg (n = 33)**	18/33	15/33	2/15	13/15
		54.55; 37.53–70.79	45.45; 29.21–62.47	13.33; 2.30–37.52	86.67; 62.48–97.70
**IIF**	**Pos (n = 90)**	68/79	11/79	28/55	27/55
		86.08; 77.09–92.45	13.92; 7.55–22.91	50.91; 37.82–63.90	49.09; 36.10–62.18
	**Neg (n = 15)**	9/13	4/13	3/5	2/5
		69.23; 41.30–89.37	30.77; 10.63–58.70	60.00; 18.24–92.65	40.00; 7.35–81.76
**MST**	**Pos (n = 74)**	65/69	4/69	21/33	12/33
		94.20; 86.61–98.13	5.80; 1.87–13.39	63.64; 46.38–78.59	36.36; 21.41–53.62
	**Neg (n = 3)**	0/3	3/3	0/2	2/2
		0.00; 0.00–63.16	100.00; 36.84–100.00	0.00; 0.00–77.64	100.00; 22.36–100.00

DS: Direct parasite search, IIF: Indirect Immunofluorescence, MST: Montenegro skin test, PCR-L: Polymerase Chain Reaction in lesion, PCR-B: Polymerase Chain Reaction in blood, Pos: Positive, Neg: Negative.

The proportions were analyzed using Mid-p exact test OpenEpi version 2.3, with confidence interval of 95%. The values are described in done number/total number, and %; 95% CI.

In 106 patients with CL, 96 had the cutaneous form. In these, PCR-L was positive in 84.44% (76/90) (95% CI; 75.84–90.86), and PCR-B in 50.00% (25/50) (95% CI; 36.34–63.66); whereas DS was positive in 65.93% (60/91) (95% CI; 55.76–75.11). In five patients, PCR-L and DS were not performed, and PCR-B was positive in 60.00% (3/5) of these patients (95% CI; 18.24–92.65). In 10 patients with the mucosal form, PCR-L was positive in 66.67% (2/3) (95% CI; 13.20–98.33), PCR-B in 60.00% (6/10) (95% CI; 29.11–85.77), and 0.00% in DS (0/2) (95% CI; 00.00–77.64).

Of 117 patients suspected of CL who did not show positive results in one or more of the conventional tests, five showed a positive PCR-L, and two others showed a positive PCR-B.

DS positivity was significantly higher in patients with lesions less than one year old, and PCR-L, PCR-B, IIF, and MST positivity did not differ over different lesion evolution times (Table 2).

**Table 2:** Comparison of PCR-L, PCR-B, DS, IIF and MST in relation to time of evolution of lesions in patients with CL.

Evolution time	PCR-L	PCR-B	DS	IIF	MST
**<3 months (n = 47)**	40/47	10/22	33/47	38/46	36/38
	85.11; 72.75–93.25	45.45; 25.88–66.16	70.21; 56.07–81.94	82.61; 69.64–91.58	94.74; 83.68–99.11
**3–12 months (n = 36)**	29/33	13/21	26/34	32/36	28/28
	87.88; 73.30–96.03	61.90; 4.23–80.49	76.47; 60.16–88.43	88.89; 75.34–96.37	100.00; 89.85–100.00
**>12 months (n = 9)**	6/8	2/6	1/8	8/9	5/6
	75.00; 38.83–95.57	33.33; 6.02–73.81	12.50; 0.63–48.03	88.89; 56.14–99.44	83.33; 40.91–99.17

DS: Direct parasite search, IIF: Indirect Immunofluorescence, MST: Montenegro skin test, PCR-L: Polymerase Chain Reaction in lesion, PCR-B: Polymerase Chain Reaction in blood, Pos: Positive, Neg: Negative.

The proportions were analyzed using Mid-p exact test OpenEpi version 2.3, with confidence interval of 95%. The values are described in done number/total number, and %; 95% CI.

The PCR-L sensitivity was similar to the MST sensitivity. The PCR-B sensitivity did not differ from the IIF sensitivity, but was significantly lower than MST and PCR-L. PCR-B showed higher specificity values and PPV than IIF, MST, and PCR-L. The best values of NPV were obtained from MST and PCR-L; there was no significant difference between the NPV of PCR-L and IIF (Table 3).

**Table 3:** Performances of PCR-L, PCR-B, IIF and MST performance for laboratory diagnosis of CL.

DS	PCR-L		PCR-B		IIF		MST	
	Pos	Neg	Pos	Neg	Pos	Neg	Pos	Neg
**Pos**	59	0	21	11	53	6	52	0
**Neg**	23	111	4	53	26	110	16	87
**S**	59/59		21/32		53/59		52/52	
	100.00; 95.05–100.00		65.63; 48.12–80.42		89.83; 80.05–95.77		100.00; 94.40–100.00	
**Sp**	111/134		53/57		110/136		87/103	
	82.84; 75.74–88.52		92.98; 83.94–97.73		80.88; 73.62–86.84		84.47; 76.49–90.52	
**PPV**	59/82		21/25		53/79		52/68	
	72.95; 61.52–80.86		84.00; 65.78–94.70		67.09; 56.18–76.76		76.47; 65.32–85.40	
**NPV**	111/111		53/64		110/116		87/87	
	100.00; 97.34–100.00		82.81; 72.10–90.62		94.83; 89.55–97.88		100.00; 96.62–100.00	

S: sensitivity, Sp: specificity, PPV: positive predictive value, NPV: negative predictive value, DS: Direct parasite search, IIF: Indirect Immunofluorescence, MST: Montenegro skin test, PCR-L: Polymerase Chain Reaction in lesion, PCR-B: Polymerase Chain Reaction in blood, Pos: Positive, Neg: Negative.

The proportions were analyzed using Mid-p exact test OpenEpi version 2.3, with confidence interval of 95%. The values of S, Sp, PPV and NPV were determined for the DS test, and they are described in done number/total number, and %; 95% CI.

## Discussion

CL is a serious public-health problem that can lead to destructive, disfiguring and disabling lesions, and even to death, mainly due to a delay in diagnosis and inadequate treatment [2], [3]. Faster, more-reliable, and more-specific methods of laboratory diagnosis are needed in order to begin treatment promptly, ensure correct species identification, and differentiate CL from other diseases with similar clinical signs [3], [23].

PCR has been evaluated in endemic areas and offers advantages over conventional tests; it is more specific, sensitive, versatile, and faster [24]. PCR has shown good sensitivity in studies on *Leishmania*, and has been used with material from culture [15], lesions [8], [10], [17], [23], [24], [25], [26], [27], blood [9], [24], [28], [29], [30], [31], and sandflies [16], [18].

The choice of primers for the PCR test for *Leishmania* is important because it influences the sensitivity of the technique, and can allow differentiation of species of the subgenera *Viannia* and *Leishmania* [30], [32]. PCR with MP3H/MP1L primers shows good sensitivity in detecting members of the subgenus *Leishmania (Viannia)*, and can detect 2 fg of DNA [32], making this technique suitable for CL diagnosis [25], [26].

PCR with GH20 and PC04 primers showed good sensitivity and can be used effectively as an internal control for human samples, excluding PCR inhibitors.

In this study, PCR-L showed high positivity (83.87%) in patients with CL, and was significantly more efficient than DS, confirming other studies [8], [24], [27]. The evaluation parameters of diagnostic tests showed 100% sensitivity and NPV in PCR-L. However, a negative PCR result with MP3H/MP1L primers does not eliminate the possibility of infection by *L. (L.) amazonensis*, which has been reported in northern Paraná [33].

PCR-B showed 51.67% positivity. Parasite DNA has been detected in blood infrequently, and other investigators have reported that PCR in blood showed low positivity [9], [31]. In this study, evaluation of the parameters for the diagnostic tests showed that PCR-B had the highest rate of specificity and PPV, compared to the other tests.

Although DS is rapid and easy to perform, it has limited application in patients without lesions or with old lesions, because the possibility that the parasite is present is inversely proportional to the age of the lesion [2]. Furthermore, DS does not distinguish the species of *Leishmania*. In this study, DS showed 64.52% positivity in CL patients; however, positivity was very low in patients with lesions older than 12 months. Other investigators have reported similar positive results, with higher rates of positivity in lesions with less than 3 months of evolution [7], [8], [34].

The IIF was positive in 85.71%, showing a good positivity rate in patients with both recent and old lesions, which is consistent with other reports [7]. However, serological methods may show cross-reactivity with Chagas’ disease [2], [6], which is common in Brazil, and show higher rates of negativity in patients with only one lesion or with lesions less than 6 months old [2].

MST has been reported to show high positivity rates [7], [8], [9], in agreement whith our finding of a positivity rate of 96.10%. However, MST is an invasive technique, may give a positive result in latent infections, and does not distinguish a past from a current infection [8], [10], [34]. This may explain the four patients who showed positive results only with the MST technique.

The majority of leishmaniasis cases in Paraná are caused by *L. (V.) braziliensis,* which has the potential to develop a mucosal form [2], [19] and can be fatal if left untreated. The occurrence of mucosal leishmaniasis ranges from 3 to 5% of cases of infection by *L. (V.) braziliensis* [3]. In Paraná, 290 cases of CL were reported in 2010, with 67 (23.10%) cases of mucosal leishmaniasis. This proportion varies by region, and in some municipalities, 100% of the cases are the mucosal form. The parasites may persist for years in the host [35], [36] and hematogenous and lymphatic dissemination can occur [28], [31], mainly by leukocytes. This is the most probable mechanism to explain the occurrence of metastatic forms (mucosal and recurrent cutaneous lesions).

Two patients who showed only a positive PCR-B had the cutaneous form and had not previously been diagnosed with CL, although they were residents of endemic areas. Other investigators have reported cases of positive results with IIF and enzyme immunoassay [37] and with PCR [28] in the blood of patients who had never had CL, but lived in endemic areas; this may explain the possibility of latent infection in asymptomatic (subclinical) carriers of *L. (V.) braziliensis*.

Five patients who showed a positive PCR-L and negative conventional tests had the cutaneous form, with active lesions lasting from 2 weeks to 2 years. Four of these patients have had CL in the past, ranging from 10 months to 8 years previously; the remaining patient, who lived in an endemic area, denied a past infection. Cases of positive PCR have been reported in samples from lesions [17], scars [38], and gingiva [39] from patients who were previously infected with and treated for *L. (V.) braziliensis*, which may explain the persistence of the infection. It must be remembered that a finding of their DNA does not imply that viable parasites are present, and that a positive PCR does not distinguish active from past infections.

CL cases (positive DS and/or PCR-L) that show a negative IIF and MST may indicate the possibility that the parasite has disseminated due to a poor host immune response [3]. In this study, five patients showed a positive PCR-L but negative IIF and MST. Such cases should be monitored, and PCR-B may be an important tool to detect possible spreading, since DS and PCR-L tend to become negative because of treatment or evolution of the lesion over time, and healing of the lesion may make these tests inviable. Investigation of *L. (V.) braziliensis* in peripheral blood has been suggested as a means of predicting a relapse [24], and for follow up and monitoring the clinical status of chemotherapy patients [30]. In 110 patients, none of the tests gave a positive result for CL, which suggests that clinical tests need to be improved; these patients returned to their physician for further evaluation.

Different types of clinical samples may vary widely in PCR sensitivity. PCR using whole blood [29], [30], [31] may show interference, resulting in low sensitivity, as also may occur with PCR using “buffy coat” samples [9], [28], [30], [31] or mononuclear cells [24], [31]. The leukocyte separation technique used in this study reduced the number of erythrocytes and other inhibitors, and also provided for the concentration of potential carrier cells of *Leishmania* and increased the PCR sensitivity. The methodology for obtaining leukocytes (EDTA/Dextran solution) effectively increased the number of these cells (4,300 leukocytes/mm^3^ of blood). Several studies have evaluated the use of PCR in the diagnosis of CL; however, ours is the first study employing leukocytes concentrated from peripheral blood obtained with dextran solution.

The use of a combination of different methodologies to improve the diagnosis of CL has been suggested [19], [40], and the choice of the methods used also depends on the evolution of the lesion or infection over time [2], [7]. However, conventional techniques do not reveal the infecting species. Patients with suspected CL must receive a definitive diagnosis, because they can progress to the potentially fatal mucosal form if left untreated. Therefore, there is a need for more sensitive methods that can be employed to follow patients and monitor their cure. At the present time, the finding of the parasite by DS is considered the only definitive diagnosis of CL [2], but this method has low sensitivity, which limits its use as a reference technique.

In view of several considerations, that: (i) the parasites can persist for years in the host; (ii) the chance of finding the parasite by DS is inversely proportional to the evolution of the lesion over time; (iii) MST is invasive; and (iv) negative IIF and MST in CL patients may suggest that the parasite has spread, it is necessary to continue research to develop more-sensitive and more-specific methods for diagnosis and evaluating the prognosis of CL. PCR-L is an alternative method for the diagnosis of CL, especially in patients with chronic lesions or who have received a specific treatment, or in patients with reinfection or a relapse of infection with *L. (V.) braziliensis*, which may evolve into a mucosal lesion. PCR-B using DNA from concentrated leukocytes showed a sensitivity comparable to that of DS, and showed a higher rate of positivity than the PCR techniques using blood that were described in other studies. This technique may be indicated in patients suspected of CL who have a negative result in conventional tests, or in patients with no lesion. Given that the detection of DNA is a reliable indication of the presence of the parasite, PCR is a useful tool for diagnosis and epidemiology of CL, and has the advantage of identifying the infecting species.
